# Supplementary material for: The functional overlap between respiration and global signal and its behavioral relevance
Source: Commun Biol. 2025 May 26;8:809. doi: 10.1038/s42003-025-08260-z (PMC12106718; doi:10.1038/s42003-025-08260-z)
Supplement: Supplementary file 1 — Supplementary Information [file 42003_2025_8260_MOESM1_ESM.pdf]

Supplementary Information

Supplementary Figures

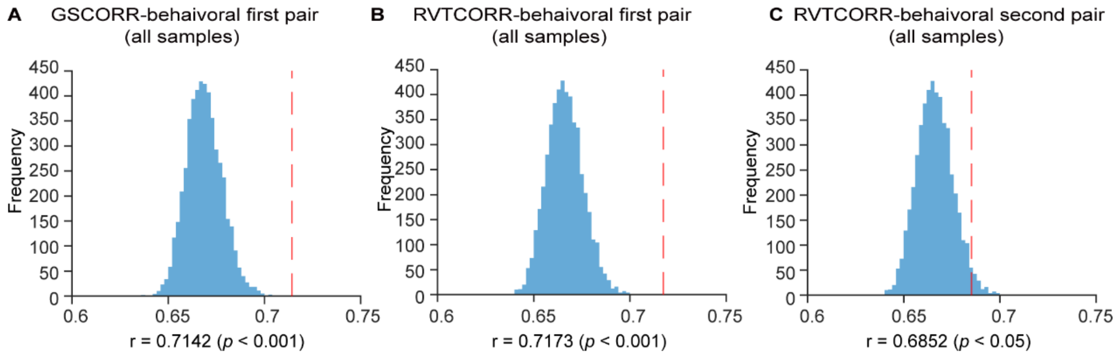

Supplementary Figure 1. The permutation test results of canonical correlation analysis (CCA) using all samples. The permutation test result for the first CCA mode in the **A.** GSCORR-behavior pair and **B.** RVTCORR-behavior pair. **C.** The permutation test result for the second CCA mode in the RVTCORR-behavior pair. The null distribution of permuted canonical correlation coefficients (blue histogram) is shown alongside the empirical coefficient (red dashed line).

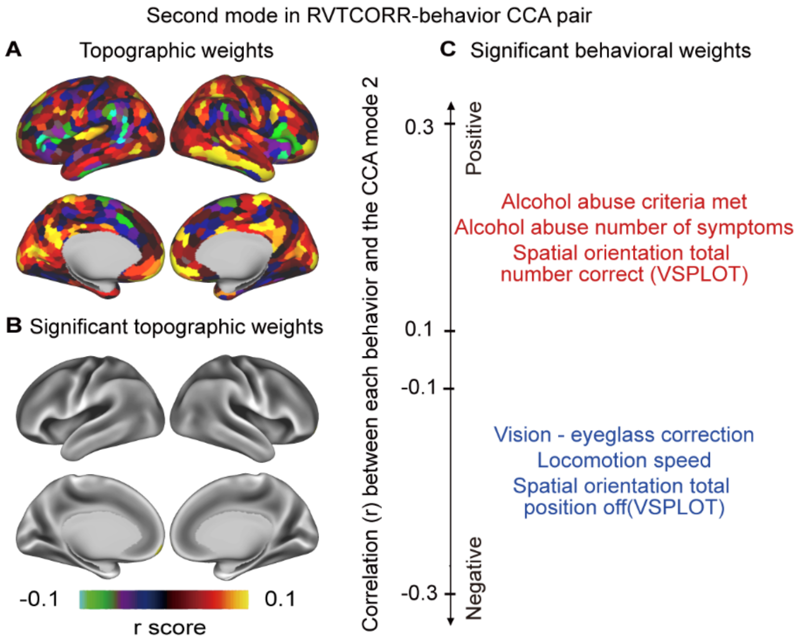

Supplementary Figure 2. Weights on the second canonical correlation analysis (CCA) mode in RVTCORR-behavior pair. **A.** RVTCORR weights associated with the topographic scores of the second CCA mode. **B.** RVTCORR weights that are significantly correlated with the topographic scores of the second CCA mode ($p < 0.001$, using 10,000 multiple permutations controlled for FWER, same for subsequent significance). **C.** Behavioral weights significantly associated with behavioral scores of the second CCA mode ($p < 0.001$). Behavioral variables colored in red and blue indicate

positive and negative weights, respectively. VSPLOT, Variable Short Penn Line Orientation Test.

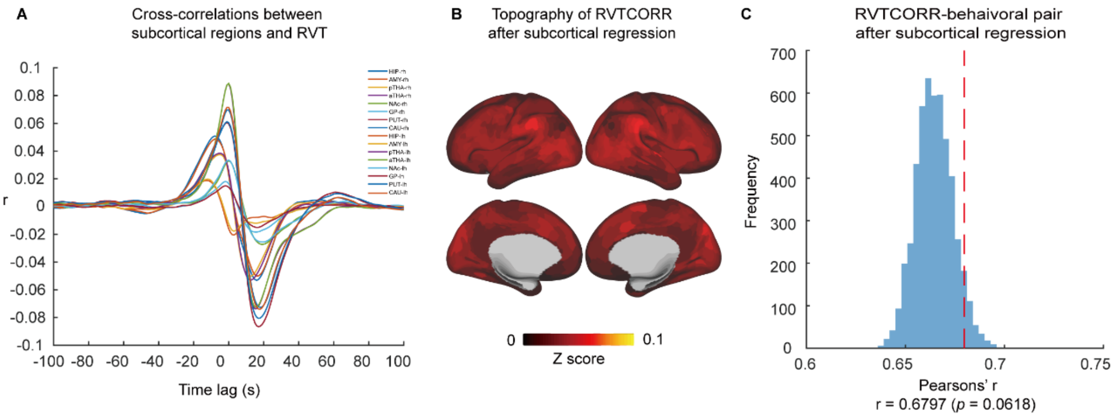

Supplementary Figure 3. Relationships between respiration and subcortical regions. **A.** Cross-correlation between subcortical regions and respiration volume per time (RVT). **B.** Group-averaged topography of RVT correlations (RVTCORR) after regressing out subcortical signals. **C.** The permutation test result for the first canonical correlation analysis (CCA) mode in the RVTCORR-behavior pair after regressing out subcortical signals. The null distribution of permuted canonical correlation coefficients (blue histogram) is shown alongside the empirical coefficient (red dashed line).

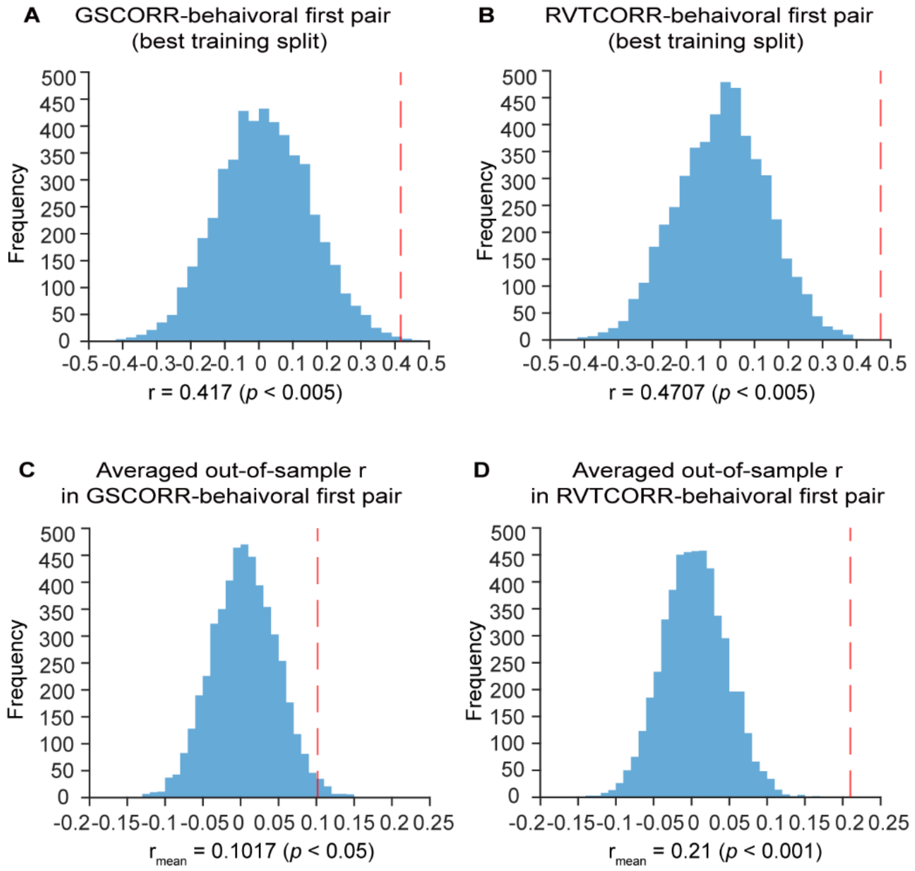

Supplementary Figure 4. The permutation test results of canonical correlation analysis (CCA) using out-of-sample correlation coefficients. The permutation test result for the first CCA mode using the best training split in the **A.** GSCORR-behavior pair and **B.** RVTCORR-behavior pair. These results are based on the training split that yielded the highest out-of-sample correlation coefficient. The permutation test result for the first CCA mode using the averaged out-of-sample r in the **C.** GSCORR-behavior pair and **D.** RVTCORR-behavior pair. For all panels, the null distribution of permuted canonical correlation coefficients (blue histogram) is shown alongside the empirical coefficient (red dashed line).

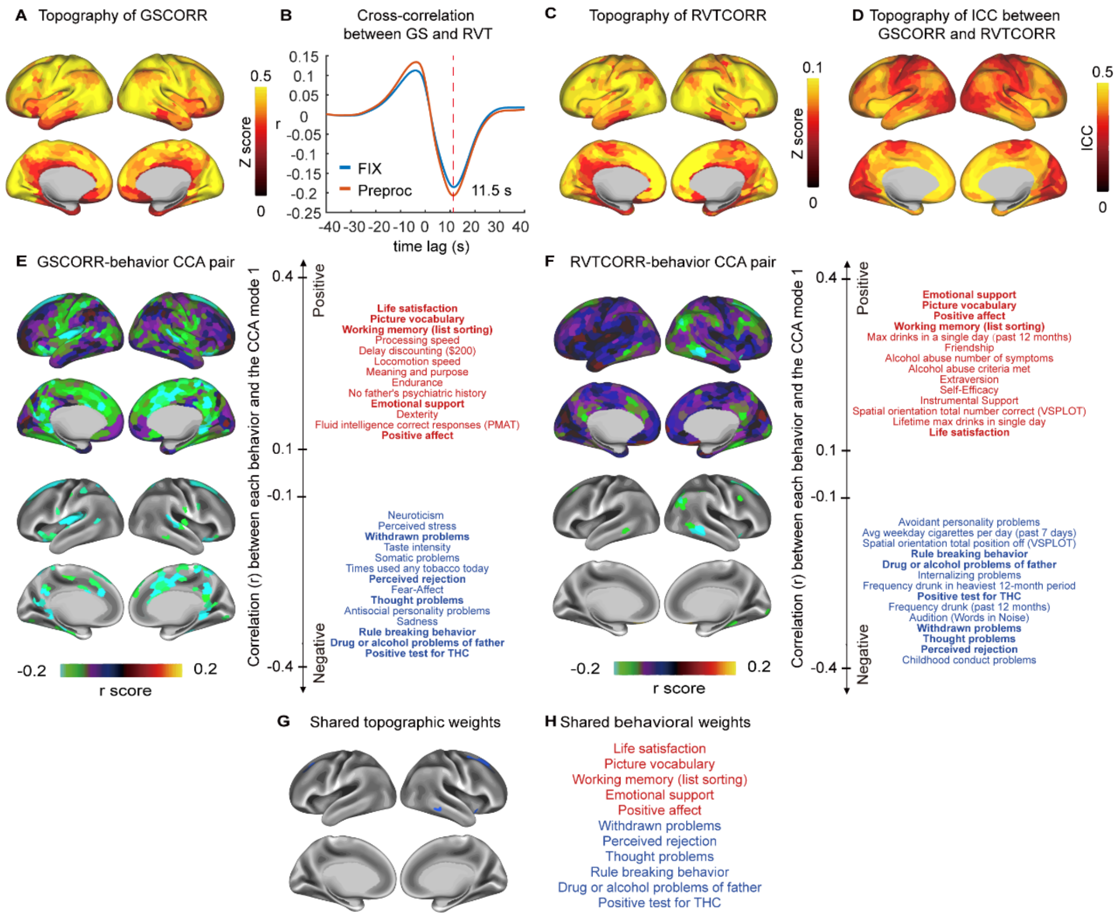

Supplementary Figure 5. Topographies, spatial similarity of global signal (GS) and respiration volume per time (RVT), and canonical correlation analysis (CCA) with behaviors based on minimal-preprocessing version. **A.** Group-averaged topography of GS correlations (GSCORR). **B.** Cross-correlation between GS and RVT in the minimal-preprocessing (orange line) and ICA-FIX (blue line) versions. The peak correlation occurs at a lag of 11.5 seconds. **C.** Group-averaged topography of RVT correlations (RVTCORR). **D.** Spatial consistency between GSCORR and RVTCORR, measured using intraclass correlation coefficient (ICC). **E.** Significant (n = 770, $p < 0.001$, using 10,000 multiple permutations controlled for FWER, same for subsequent significance) weights on the first CCA mode in the GSCORR-behavior pair. **F.** Significant ($p < 0.001$) weights on the first CCA mode in the RVTCORR-behavior pair. Bold font implies shared behavioral weights between the GSCORR-behavior and RVTCORR-behavior pairs. **G.** Overlap of significant ($p < 0.001$) topographic weights. **H.** Overlap of significant ($p < 0.001$) behavioral weights. PMAT, Penn Progressive Matrices Test; THC, Δ^9-tetrahydrocannabinol.

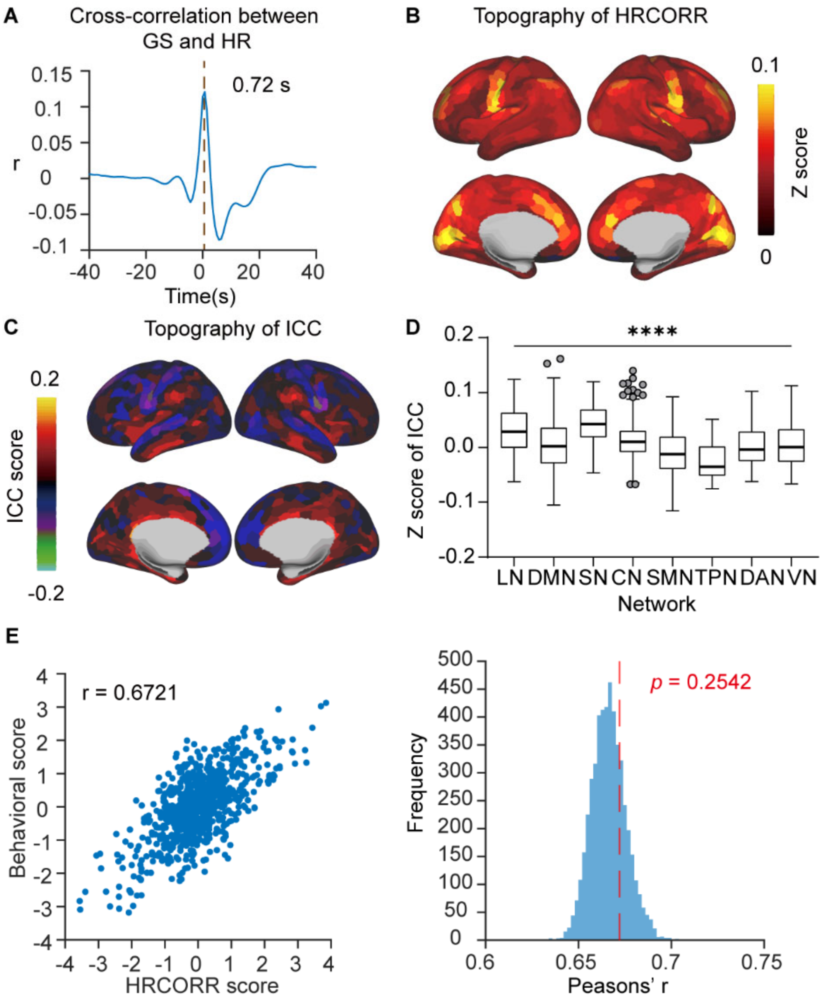

Supplementary Figure 6. Topography, spatial similarity of heart rate (HR) and canonical correlation analysis (CCA) with behavioral measures based on minimal-preprocessing version. **A.** Cross-correlation between global signal (GS) and HR. **B.** Group-averaged topography of heart rate correlations (HRCORR). **C.** Spatial consistency between GSCORR and HRCORR, quantified using the intraclass correlation coefficient (ICC). **D.** Boxplot showing the Fisher's z-transformed ICC between GSCORR and HRCORR grouped by networks. Statistical comparisons across networks were performed using the Kruskal–Wallis test. **E.** CCA results between HRCORR and behavioral measures. Left panel: scatterplot of behavioral scores versus HRCORR scores of the first CCA mode, where each point represents one participant. Right panel: null distribution of permuted canonical correlation coefficients (blue histogram) and the empirical canonical correlation coefficient (red dashed line). **** $p < 0.0001$. LN, limbic network; DMN, default mode network; SN, salience network; CN, control network; SMN, somatomotor network; TPN, temporal parietal network; DAN, dorsal attention network; VN, visual network.

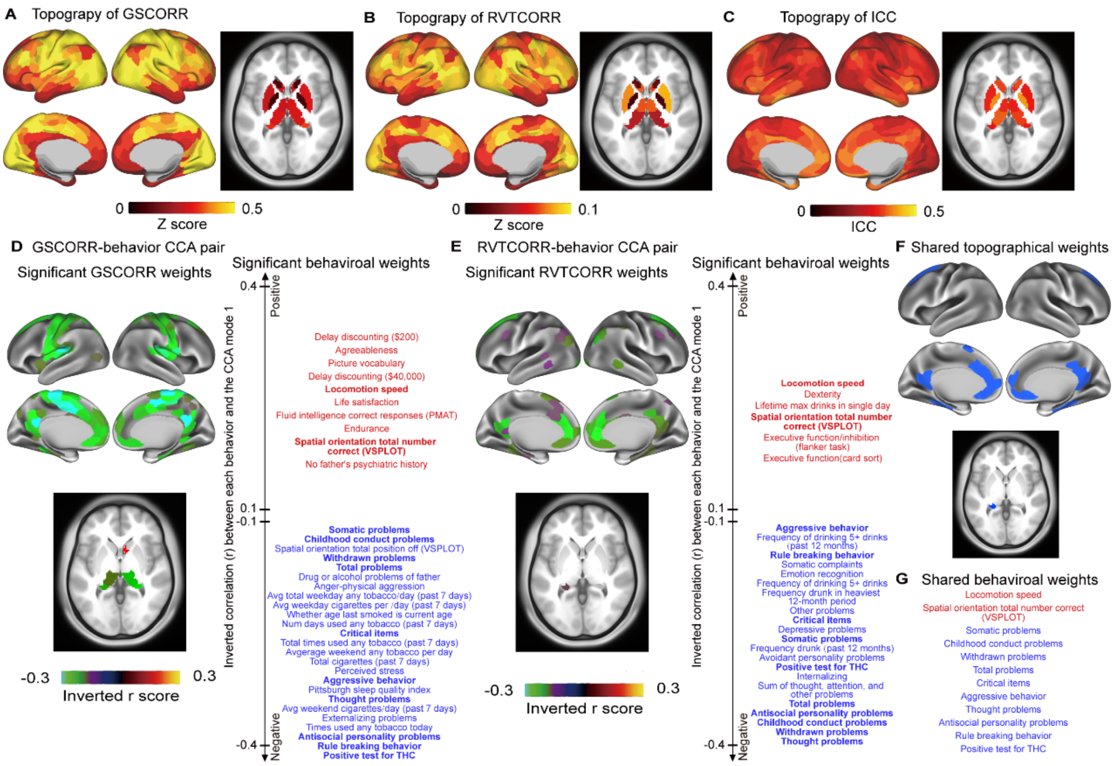

Supplementary Figure 7. Topographies, spatial similarity of global signal (GS) and respiration volume per time (RVT), and canonical correlation analysis (CCA) with behavior measures using a template that includes subcortical regions. **A.** Group-averaged topography of GS correlations (GSCORR). **B.** Group-averaged topography of RVT correlations (RVTCORR). **C.** Spatial consistency between GSCORR and RVTCORR, measured using intraclass correlation coefficient (ICC). **D.** Significant ($n = 770$, $p < 0.001$, using 10,000 multiple permutations controlled for FWER, same for subsequent significance) weights on the first CCA mode in the GSCORR-behavior pair. **E.** Significant ($p < 0.001$) weights on the first CCA mode in the RVTCORR-behavior pair. Bold font implies shared behavioral weights between the GSCORR-behavior and RVTCORR-behavior pairs. **F.** Overlap of significant ($p < 0.001$) topographic weights. **G.** Overlap of significant ($p < 0.001$) behavioral weights. The signs of weights in the RVTCORR-behavior and GSCORR-behavior pairs were inverted for visualization. The weights displayed here are significantly ($p < 0.001$) positively (red) or negatively (blue) associated with individual behavioral or topographic scores in both sign-inverted pairs. VSPLOT, Variable Short Penn Line Orientation Test; PMAT, Penn Progressive Matrices Test; THC,Δ^9-tetrahydrocannabinol.

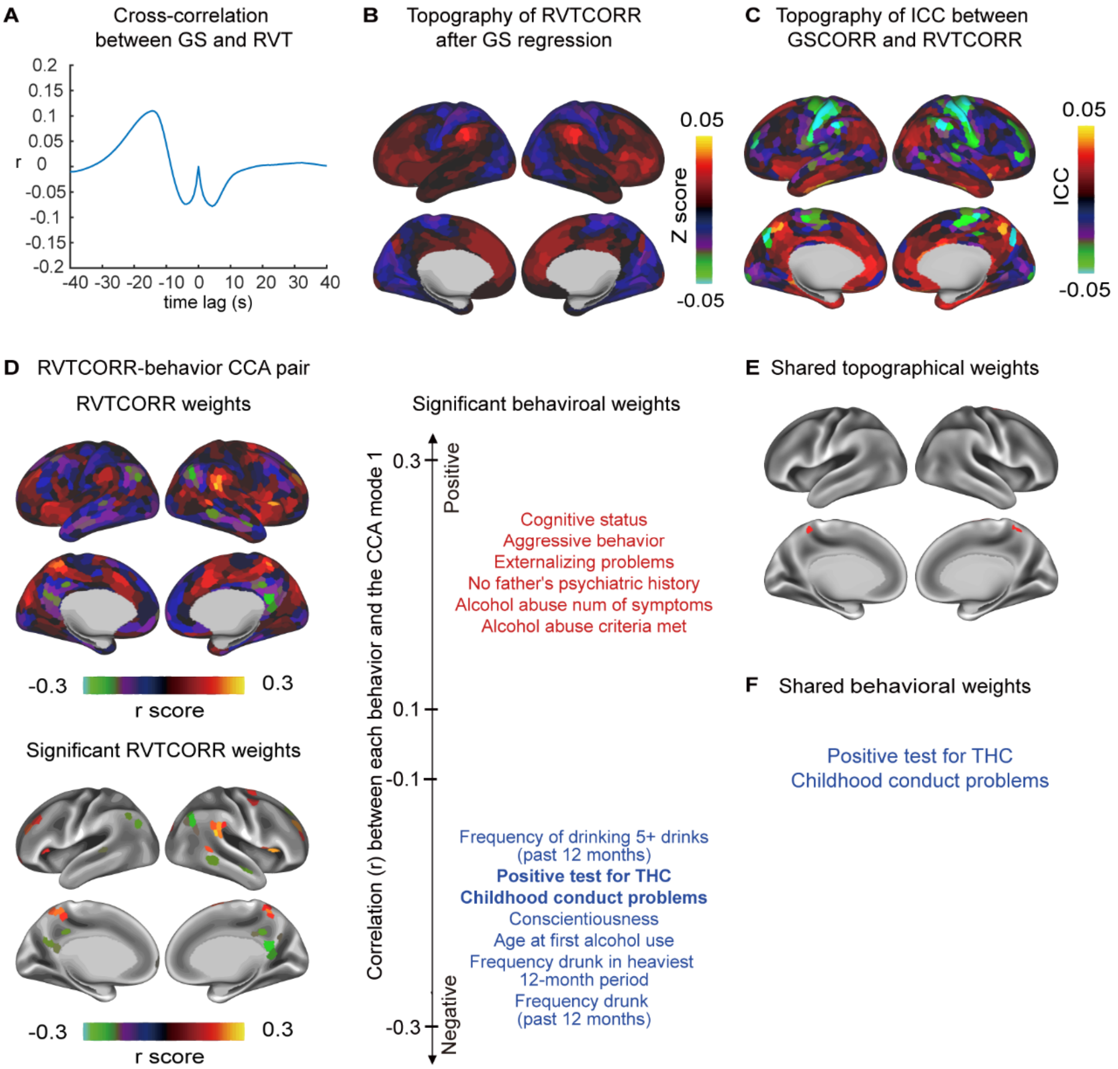

Supplementary Figure 8. Cross-correlation, topographies, spatial similarity of respiration volume per time (RVT), and canonical correlation analysis (CCA) with behavior measures, after regressing out global signal (GS) from RVT. **A.** Cross-correlation between GS and residual RVT after regression of GS. **B.** Group-averaged topography of RVT correlations (RVTCORR). **C.** Spatial consistency between GSCORR and ENVCORR, measured using intraclass correlation coefficient (ICC). **D.** Significant (n = 770, p < 0.001, using 10,000 multiple permutations controlled for FWER, same for subsequent significance) weights on the first CCA mode in the RVTCORR-behavior pair. Bold font implies shared behavioral weights between the RVTCORR-behavior pair after GS regression and the original GSCORR-behavior pair. **E.** Overlap of significant (p < 0.001) topographic weights. **F.** Overlap of significant (p < 0.001) behavioral weights. The weights displayed here are significantly (p < 0.001) positively (red) or negatively (blue) associated with individual behavioral or topographic scores in sign-inverted GSCORR-behavior pair and the RVTCORR-behavior pair. THC, Δ^9-tetrahydrocannabinol.

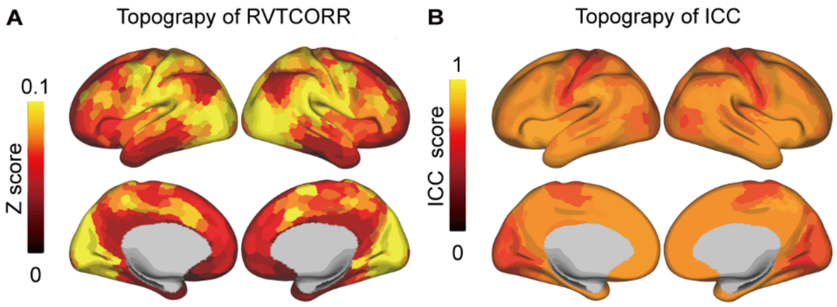

Supplementary Figure 9. Topographies based on individually optimized cross-correlation time lag. **A.** Group-averaged topography of respiration volume per time correlations (RVTCORR) using the cross-correlation lag that maximized individual-level correlation. **B.** Spatial consistency between RVTCORR based on individual versus group delays, measured using the intraclass correlation coefficient (ICC).

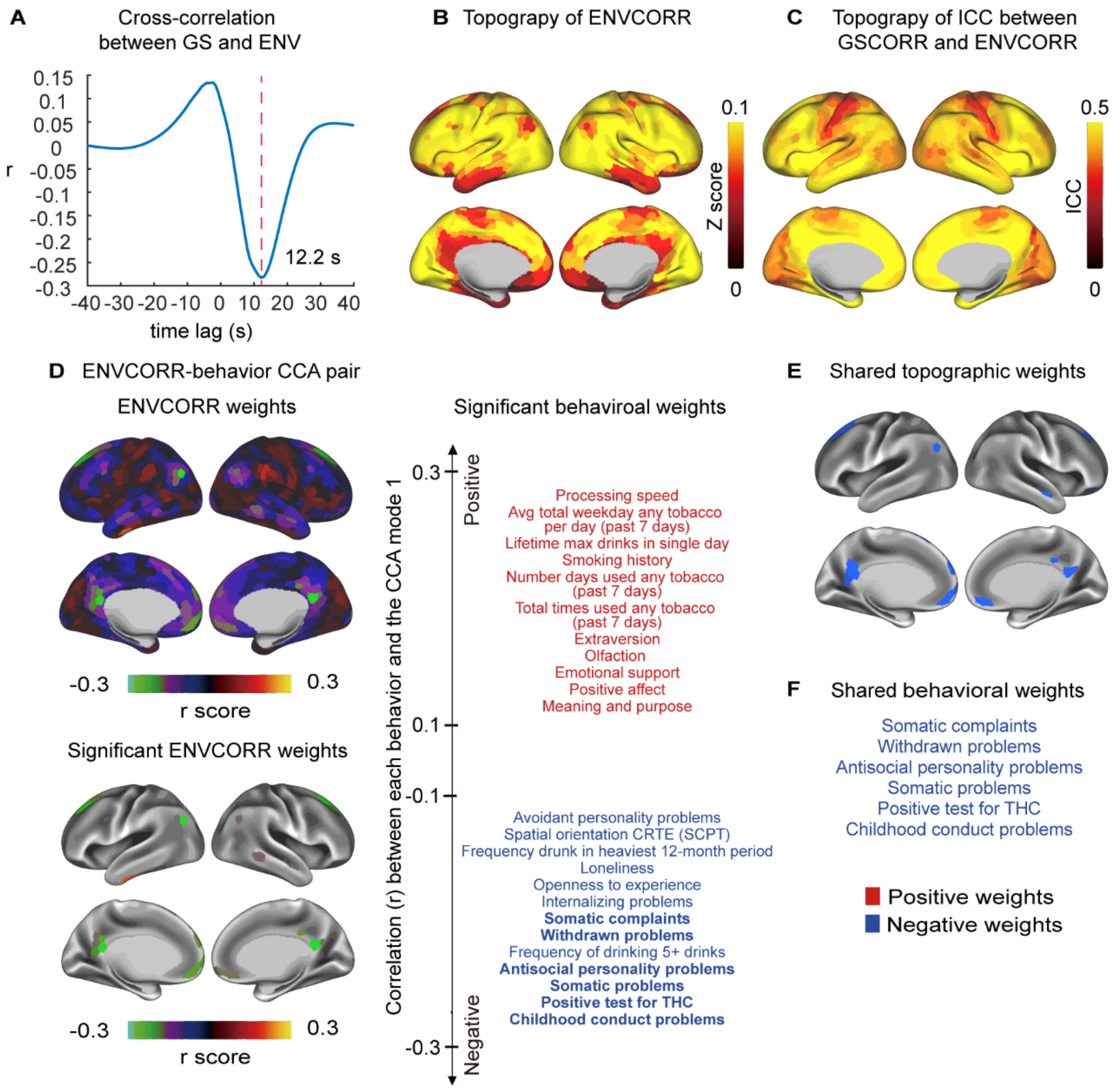

Supplementary Figure 10. Cross-correlation, topographies, spatial similarity of envelope of the waveform (ENV), and canonical correlation analysis (CCA) with behavioral measures. **A.** Cross-correlation between global signal (GS) and ENV. **B.** Group-averaged topography of ENV correlations (ENVCORR). **C.** Spatial consistency between GSCORR and ENVCORR, measured using the intraclass correlation coefficient (ICC). **D.** Significant (n = 770, p < 0.001, using 10,000 multiple permutations controlled for FWER, same for subsequent significance) weights on the first CCA mode in the ENVCORR-behavior pair. Bold font implies shared behavioral weights between the ENVCORR-behavior and GSCORR-behavior pairs. **E.** Overlap of significant (p < 0.001) topographic weights. **F.** Overlap of significant (p < 0.001) behavioral weights. The weights displayed here are significantly (p < 0.001) positively (red) or negatively (blue) associated with individual behavioral or topographic scores in sign-inverted GSCORR-behavior pair and the ENVCORR-behavior pair. CRTE, median reaction time divided by expected number of clicks for correct. SCPT, Short Penn Continuous Performance Test; THC, Δ^9-tetrahydrocannabinol.

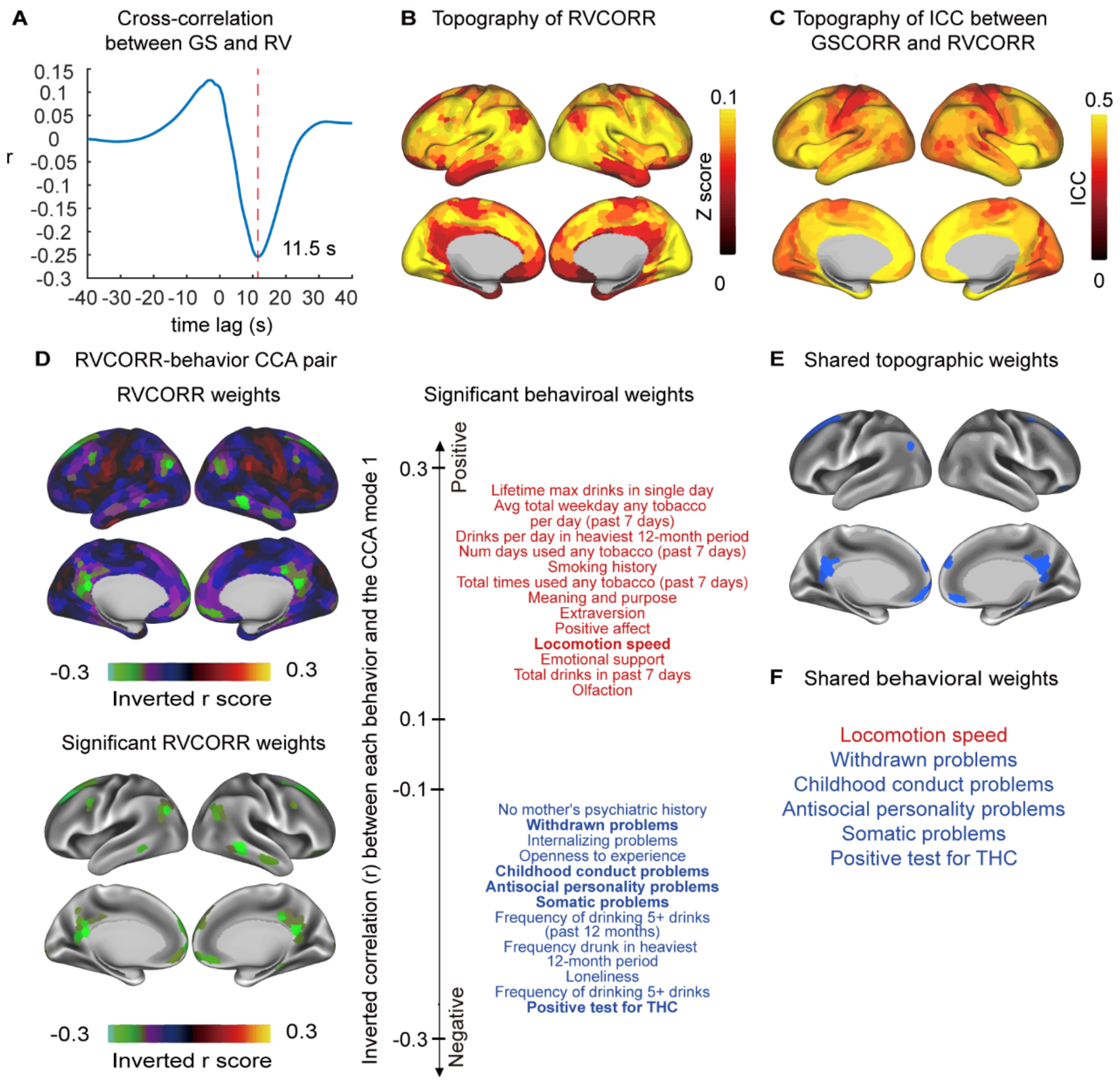

Supplementary Figure 11. Cross-correlation, topographies, spatial similarity of respiratory variation (RV), and canonical correlation analysis (CCA) with behavioral measures. **A.** Cross-correlation between GS and RV. **B.** Group-averaged topography of RV (RVCORR). **C.** Spatial consistency between GSCORR and RVCORR, measured using the intraclass correlation coefficient (ICC). **D.** Significant (n = 770, $p < 0.001$, using 10,000 multiple permutations controlled for FWER, same for subsequent significance) weights for the first CCA mode in the RVCORR-behavior pair. Bold font implies shared behavioral weights between the RVCORR-behavior and GSCORR-behavior pairs. The signs of weights were inverted for visualization. **E.** Overlap of significant ($p < 0.001$) topographic weights. **F.** Overlap of significant ($p < 0.001$) behavioral weights. The signs of weights in the GSCORR-behavior and RVCORR-behavior pairs were inverted for visualization. The weights displayed here are significantly ($p < 0.001$) positively (red) or negatively (blue) associated with individual behavioral or topographic scores in both sign-inverted pairs. THC, Δ^9 -tetrahydrocannabinol.

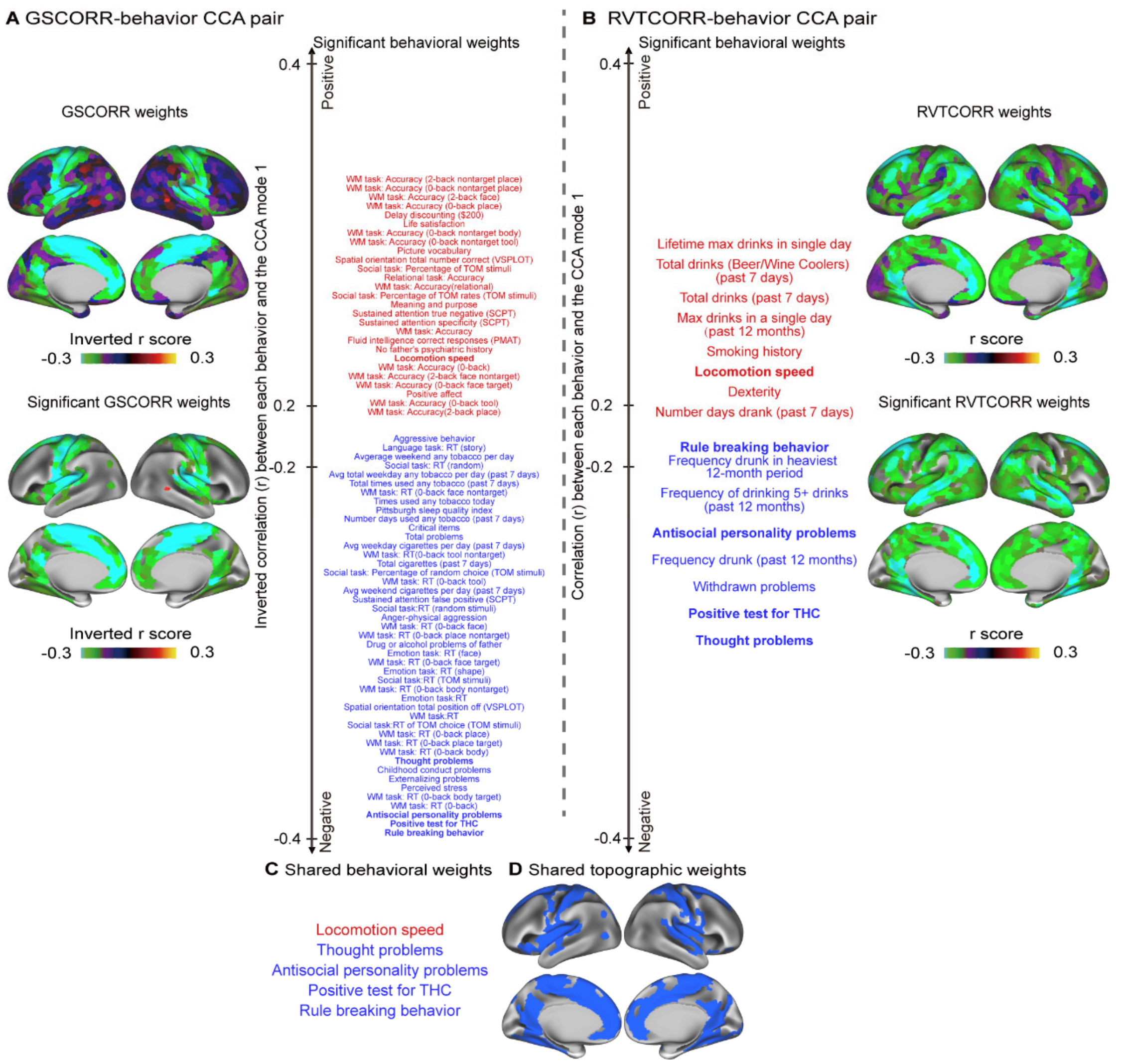

Supplementary Figure 12. Behavioral and topographic overlaps of the first CCA modes in the GSCORR-behavior and RVTCORR-behavior pairs, using behavioral measures including task-related variables. **A.** Significant (n = 770, $p < 0.001$, using 10,000 multiple permutations controlled for FWER, same for subsequent significance) weights on the first CCA mode in the GSCORR-behavior pair. **B.** Significant ($p < 0.001$) weights on the first CCA mode in the RVTCORR-behavior pair. Bold font implies shared behavioral weights between the GSCORR-behavior and RVTCORR-behavior pairs. **C.** Significant ($p < 0.001$, using 10,000 multiple permutations controlled for FWER, same for subsequent significance) behavioral weights. **D.** Overlap of significant ($p < 0.001$) topographic weights. The signs of weights in the GSCORR-behavior pairs were inverted for visualization. The weights displayed here are significantly ($p < 0.001$) positively (red) or negatively (blue) associated with individual behavioral or topographic scores in sign-inverted GSCORR-behavior pair and the RVTCORR-behavior pair. SCPT, Short Penn Continuous Performance Test; VSPLOT, Variable Short Penn Line Orientation Test; PMAT, Penn Progressive Matrices Test; TOM, Theory of Mind; THC, Δ^9-tetrahydrocannabinol.

Supplementary Note 1: Behavioral measures exclusion

We did a similar data exclusion and data reduction for the original set of 478 behavioral measures like previous study[1].

We first identified 11 behavioral measures as confounds, and regressed them out from the data. These measures included: acquisition reconstruction software version; gender; age; weight; height; BMI; systolic blood pressure; diastolic blood pressure; Hemoglobin A1cmeasured in blood; the cube-root of total brain volume (including ventricles); the cube-root of total intracranial volume.

We then identified 105 bad variables that that were quantitatively poor measures including having 100 standard deviations above the median, fewer than half valid values (i.e., 500) or same values exceeding 95% of the data.

Next, we excluded measures that were not of interest for the current analysis: T1-weighted structural supplied variables; participant ID; handedness; employment status; income level; education level; race and ethnicity; "Is the participant in college?"; "Is the participant in a live-in relationship?"; "Is the participant born in Missouri?"; BMI-related questions; thyroid/hypothyroid/endocrine onset measures; menstruation-related measures.

Finally, we excluded redundant variables in cognitive task and substance abuse, keeping minor measures: fluid intelligence secondary measures of total skipped tests and reaction time; all minor delayed discounting measures except for area-under-the-curve (AUC) for $200 and $40,000; minor visual contrast sensitivity measure of error count; alcohol use 7-day self-report expect for: total drinks /number days drank in past 7 days

(beer/wine cooler/wine/hard liquor); average total weekend drinks/day in past 7 days (wine/hard liquor); all tobacco used and dependence questions except for: times smoked any tobacco today; number of days smoked any tobacco in 7 days; total times smoked any tobacco/ cigarettes in past 7 days; average total weekday/weekend any tobacco/cigarettes per day in past 7 days; tobacco use and dependence except for smoking history and whether age last smoked is current age. For variables that included raw and adjusted scores, we kept the raw scores and excluded the adjusted scores because we had regressed out age and gender.

The above exclusion procedure resulted in a 119 behavioral measure that were fed into CCA. A list of employed behavioral measures is provided in Supplementary data 7.

THEOREM. Flipping the signs of canonical loadings for both X and Y does not affect the magnitude of canonical correlation.

Proof. Let X and Y be column-mean-centered matrices of dimensions $N \times D_{\mathrm{X}}$ and $N \times D_Y$, respectively. The canonical weights for X and Y are denoted as A and B, and the canonical variables are defined as:

$$P = XA, Q = YB$$

Suppose we flip the signs of both the canonical weights of X and Y

$$A^{'} = -A, B^{'} = -B$$

The new canonical variables P' and Q' are then given by:

$$P^{'} = XA^{'} = X(-A) = -XA = -P$$

$$Q^{'} = YB^{'} = Y(-B) = -YB = -Q$$

Thus, both P and Q have their signs reversed. Next, we examine the canonical correlation between the new canonical variables P' and Q'. The canonical correlation is defined as:

$$\rho_{P'Q'} = \frac{\left(cov\left(P', Q'\right)\right)}{sqrt\left(var\left(P'\right)var\left(Q'\right)\right)} = \frac{\left(cov(-P, -Q)\right)}{sqrt\left(var(-P)var(-Q)\right)}$$

We have:

$$Cov(-P, -Q) = Cov(P, Q), Var(-P) = Var(P), Var(-Q) = Var(Q)$$

Therefore, we conclude that:

$$\rho_{P'Q'} = \frac{\left(cov\left(P', Q'\right)\right)}{sqrt\left(var\left(P'\right)var\left(Q'\right)\right)} = \rho_{PQ}$$

This implies that although the signs of the canonical weights have been flipped, the magnitude of the canonical correlation remains unchanged.

Supplementary References

1. Smith, S. M. et al. A positive-negative mode of population covariation links brain connectivity, demographics and behavior. Nature Neuroscience 18, 1565-1567 (2015). https://doi.org/10.1038/nn.4125
