# Supplementary material for: The functional overlap between respiration and global signal and its behavioral relevance
Source: Commun Biol. 2025 May 26;8:809. doi: 10.1038/s42003-025-08260-z (PMC12106718; doi:10.1038/s42003-025-08260-z)
Supplement: Supplementary file 2 — Description of Additional Supplementary Files [file 42003_2025_8260_MOESM2_ESM.docx]

Description of Additional Supplementary Files

**File Name:** Supplementary Data 1

**Description:** Source data for Figure 1. Worksheet 1 (Tab name: Fig1A_GSCORR): Data related to Figure 1A; Group-averaged topography of GS correlations (GSCORR). Worksheet 2 (Tab name: Fig1B_r): Data related to Figure 1B; Cross-correlation values between the global signal and respiration volume per time. Worksheet 3 (Tab name: Fig1B_RVTCORR): Data related to Figure 1B; Group-averaged topography of RVT correlations (RVTCORR). Worksheet 3 (Tab name: Fig1C_ICC): Data related to Figure 1C; Intraclass correlation coefficient between GSCORR and RVTCORR. Worksheets 5-12: Data related to Figure 1D; ROIwised Fisher's z-transformed intraclass correlation coefficient (ICC) between GSCORR and RVTCORR grouped by networks (each ICC within each Page 8 of 25 network is stored in one tab). LN: limbic network; DMN: default mode network; SN: salience network; CN: control network; SMN: somatomotor network; TPN: temporal-parietal network; DAN: dorsal attention network; VN: visual network.

**File Name:** Supplementary Data 2

**Description:** Source data for Figure 2. Worksheet 1 (Tab name: Fig2A): Data related to Figure 2A-B; GSCORR weights from the first mode of the GSCORR-behavior CCA pair; Contains three columns: Parcel names, corresponding weight values, and significance levels. Worksheet 2 (Tab name: Fig2C): Data related to Figure 2C; The ratio of significant ROIs to total ROIs within each network. Worksheet 3 (Tab name: Fig2D): Data related to Figure 2D; Canonical variables from the first mode of the GSCORR-behavior CCA pair; Contains three columns: CCA behavioral scores (Y-axis), CCA GSCORR scores (X-axis), and thought problems scores (color-coded dot). Worksheet 4 (Tab name: Fig2E): Data related to Figure 2E; Behavioral weights from the first mode of the GSCORR-behavior CCA pair; Contains three columns: Names of behavioral measure, corresponding weight values, and significance levels.

**File Name:** Supplementary Data 3

**Description:** Source data for Figure 3. Worksheet 1 (Tab name: Fig3A): Data related to Figure 3A-B; RVTCORR weights from the first mode of the RVTCORR-behavior CCA pair; Contains three columns: Parcel names, corresponding weight values, and significance levels. Worksheet 2 (Tab name: Fig3C): Data related to Figure 3C; The ratio of significant ROIs to total ROIs within each network. Worksheet 3 (Tab name: Fig3D): Data related to Figure 3D; Canonical variables from the first mode of the RVTCORR-behavior CCA pair; Contains three columns: CCA behavioral scores (Y-axis), CCA RVTCORR scores (X-axis), and thought problems scores (color-coded dot). Worksheet 4 (Tab name: Fig3E): Data related to Figure 3E; Behavioral weights from the first mode of the RVTCORR-behavior CCA pair; Contains three columns: Names of behavioral measure, corresponding weight values, and significance levels.

**File Name:** Supplementary Data 4

**Description:** Source data for Figure 4. Worksheet 1 (Tab name: Fig4C): Data related to Figure 4C; Behavioral weights from the first mode of the GSCORR–behavior CCA pair after regressing out RVTCORR from GSCORR. Contains two columns: CCA behavioral scores and CCA GSCORR scores. Page 10 of 25 Worksheet 2 (Tab name: Fig4D): Data related to Figure 4D; GSCORR weights from the first mode of the GSCORR–behavior CCA pair after regressing out RVTCORR from GSCORR. Contains three columns: Parcel names, corresponding weight values, and significance levels.

**File Name:** Supplementary Data 5

**Description:** Source data for Figure 5. Worksheet 1 (Tab name: Fig5Atrain): Data related to Figure 5A; Canonical variables from the first mode of GSCORR-behavior CCA for the training set; Contains two columns: CCA behavioral scores and CCA GSCORR scores. Worksheet 2 (Tab name: Fig5Atest): Data related to Figure 5A; Canonical variables from the first mode of GSCORR-behavior CCA for the testing set; Contains two columns: CCA behavioral scores and CCA GSCORR scores. Worksheet 3 (Tab name: Fig5A_GSCORR): Data related to Figure 5A; GSCORR weights from the first mode of the GSCORR-behavior CCA pair; Contains three columns: Parcel names, corresponding weight values, and significance levels. Worksheet 4 (Tab name: Fig5A_beh): Data related to Figure 5A; Behavioral weights from the first mode of the GSCORR-behavior CCA pair; Contains three columns: Names of behavioral measure, corresponding weight values, and significance levels. Page 11 of 25 Worksheet 5 (Tab name: Fig5Btrain): Data related to Figure 5B; Canonical variables from the first mode of RVTCORR-behavior CCA for the training set; Contains two columns: CCA behavioral scores and CCA RVTCORR scores. Worksheet 6 (Tab name: Fig5Btest): Data related to Figure 5B; Canonical variables from the first mode of RVTCORR -behavior CCA for the testing set; Contains two columns: CCA behavioral scores and CCA RVTCORR scores. Worksheet 7 (Tab name: Fig5B): Data related to Figure 5B; RVTCORR weights from the first mode of the GSCORR-behavior CCA pair; Three columns of data: Parcel names, corresponding weight values, and significance levels. Worksheet 8 (Tab name: Fig5B): Data related to Figure 5B; Behavioral weights from the first mode of the RVTCORR-behavior CCA pair; Contains three columns: Names of behavioral measure, corresponding weight values, and significance levels.

**File Name:** Supplementary Data 6

**Description:** Source data for Figure 6. Worksheet 1 (Tab name: Fig6A): Data related to Figure 6A; Cross-correlation value between the global signal and heart rate. Worksheet 2 (Tab name: Fig6B): Data related to Figure 6B; Group-averaged topography of HR correlations (HRCORR). Page 12 of 25 Worksheet 3 (Tab name: Fig6C): Data related to Figure 6C; Intraclass correlation coefficient between GSCORR and HRCORR. Worksheets 4-11: Data related to Figure 6D; ROIwised Fisher's z-transformed intraclass correlation coefficient (ICC) between GSCORR and HRCORR grouped by networks (each ICC within each network is stored in one tab). LN: limbic network; DMN: default mode network; SN: salience network; CN: control network; SMN: somatomotor network; TPN: temporal-parietal network; DAN: dorsal attention network; VN: visual network. Worksheet 12 (Tab name: Fig6E): Data related to Figure 6E; Canonical variables from the first mode of the HRCORR-behavior CCA pair; Contains three columns: CCA behavioral scores (Y-axis) and CCA HRCORR scores (X-axis). Worksheet 13 (Tab name: Fig6F_rnull): Data related to Figure 6F; Null distribution of permuted canonical correlation coefficients of the HRCORRbehavior CCA pair. Worksheet 14 (Tab name: Fig6F_rvalue): Data related to Figure 6F; Empirical canonical correlation coefficient of the HRCORR-behavior CCA pair.

**File Name:** Supplementary Data 7

**Description:** Large table containing behavioral measures and their corresponding functions (Tab name: Behavioral measures) and large table Page 13 of 25 containing additional task measures and their corresponding functions (Tab name: Addition task measures).
