# Supplementary material for: The functional overlap between respiration and global signal and its behavioral relevance
Source: Commun Biol. 2025 May 26;8:809. doi: 10.1038/s42003-025-08260-z (PMC12106718; doi:10.1038/s42003-025-08260-z)
Supplement: Supplementary file 10 — Reporting Summary [file 42003_2025_8260_MOESM10_ESM.pdf]

nature portfolio

Corresponding author(s): COMMSBIO-24-3094B

Last updated by author(s): May 3, 2025

Reporting Summary

Nature Portfolio wishes to improve the reproducibility of the work that we publish. This form provides structure for consistency and transparency in reporting. For further information on Nature Portfolio policies, see our Editorial Policies and the Editorial Policy Checklist.

Statistics

For all statistical analyses, confirm that the following items are present in the figure legend, table legend, main text, or Methods section.

n/a	Confirmed	
☐	☒	The exact sample size (*n*) for each experimental group/condition, given as a discrete number and unit of measurement
☐	☒	A statement on whether measurements were taken from distinct samples or whether the same sample was measured repeatedly
☐	☒	The statistical test(s) used AND whether they are one- or two-sided<br>*Only common tests should be described solely by name; describe more complex techniques in the Methods section.*
☐	☒	A description of all covariates tested
☐	☒	A description of any assumptions or corrections, such as tests of normality and adjustment for multiple comparisons
☐	☒	A full description of the statistical parameters including central tendency (e.g. means) or other basic estimates (e.g. regression coefficient) AND variation (e.g. standard deviation) or associated estimates of uncertainty (e.g. confidence intervals)
☐	☒	For null hypothesis testing, the test statistic (e.g. *F*, *t*, *r*) with confidence intervals, effect sizes, degrees of freedom and *P* value noted<br>*Give P values as exact values whenever suitable.*
☒	☐	For Bayesian analysis, information on the choice of priors and Markov chain Monte Carlo settings
☒	☐	For hierarchical and complex designs, identification of the appropriate level for tests and full reporting of outcomes
☐	☒	Estimates of effect sizes (e.g. Cohen's *d*, Pearson's *r*), indicating how they were calculated

Our web collection on statistics for biologists contains articles on many of the points above.

Software and code

Policy information about availability of computer code

Data collection	Data were collected from the Human Connectome Project (HCP) S1200 Release.
Data analysis	Data analysis was implemented using Workbench and custom scripts in MATLAB 2020b.

For manuscripts utilizing custom algorithms or software that are central to the research but not yet described in published literature, software must be made available to editors and reviewers. We strongly encourage code deposition in a community repository (e.g. GitHub). See the Nature Portfolio guidelines for submitting code & software for further information.

Data

Policy information about availability of data

All manuscripts must include a data availability statement. This statement should provide the following information, where applicable:

- Accession codes, unique identifiers, or web links for publicly available datasets
- A description of any restrictions on data availability
- For clinical datasets or third party data, please ensure that the statement adheres to our policy

The MRI and behavioral datasets including open and restricted data used in this study are available in the Human Connectome Project (S1200 release) repository, https://www.humanconnectome.org/study/hcp-young-adult/document/1200-subjects-data-release.

Research involving human participants, their data, or biological material

Policy information about studies with human participants or human data. See also policy information about sex, gender (identity/presentation), and sexual orientation and race, ethnicity and racism.

Reporting on sex and gender	A total of 770 participants (416 females, 354 males) were included. Sex was used as a confounding variable in the main analysis.
Reporting on race, ethnicity, or other socially relevant groupings	This study did not address the use of race, ethnicity, or other socially relevant groupings.
Population characteristics	The sample consisted of 770 participants from HCP S1200 Release. These participants were from 368 families, including 89 monozygotic twins and 53 dizygotic twins.
Recruitment	Participants were recruited from the HCP dataset.
Ethics oversight	Informed consent was obtained from all participants. The study has been conducted in accordance with Declaration of Helsinki.

Note that full information on the approval of the study protocol must also be provided in the manuscript.

Field-specific reporting

Please select the one below that is the best fit for your research. If you are not sure, read the appropriate sections before making your selection.

☐ Life sciences ☒ Behavioural & social sciences ☐ Ecological, evolutionary & environmental sciences

For a reference copy of the document with all sections, see nature.com/documents/nr-reporting-summary-flat.pdf

Behavioural & social sciences study design

All studies must disclose on these points even when the disclosure is negative.

Study description	Canonical correlation analysis was used to model the relationship between global signal (GS) topography and behaviors, as well as the relationship between respiration topography and behaviors.
Research sample	770 participants (416 females, 354 males).
Sampling strategy	The study only involved retrospective analyses.
Data collection	The sample consisted of 770 participants (354 males; age range: 22-37 years) from the Human Connectome Project (HCP) S1200 release. Resting-state functional MRI data were obtained from the HCP dataset, along with the respiration signals, cardiac signals and behavioral data.
Timing	Data collection ranged from 2012 to 2015.
Data exclusions	We excluded data from original 1100 participants based on the exclusion criteria indicated as follows: (1) missing entire rs-fMRI time series for any run (n = 82); (2) insufficient rs-fMRI time series for any run (n = 15); (3) loss of physiological recordings (n = 87); (4) insufficient numbers of triggers in physiological recordings (n = 120); (5) inability to perform reliable peak detection of the respiratory traces or reliable peak detection of the cardiac trace (n = 24); (6) participants without family structure (n = 2). The final sample comprised 770 participants.
Non-participation	This study did not provide information about participant dropout.
Randomization	No randomization was applied in this study. Confounding variables, including acquisition reconstruction software version, gender, age, weight, height, BMI, systolic blood pressure, diastolic blood pressure, Hemoglobin A1c measured in blood, the cube-root of total brain volume (including ventricles) and the cube-root of total intracranial volume, were regressed out from the data prior to the main analysis.

Reporting for specific materials, systems and methods

We require information from authors about some types of materials, experimental systems and methods used in many studies. Here, indicate whether each material, system or method listed is relevant to your study. If you are not sure if a list item applies to your research, read the appropriate section before selecting a response.

Materials & experimental systems

n/a	Involved in the study
☒	☐ Antibodies
☒	☐ Eukaryotic cell lines
☒	☐ Palaeontology and archaeology
☒	☐ Animals and other organisms
☒	☐ Clinical data
☒	☐ Dual use research of concern
☒	☐ Plants

Methods

n/a	Involved in the study
☒	☐ ChIP-seq
☒	☐ Flow cytometry
☐	☒ MRI-based neuroimaging

Plants

Seed stocks

Report on the source of all seed stocks or other plant material used. If applicable, state the seed stock centre and catalogue number. If plant specimens were collected from the field, describe the collection location, date and sampling procedures.

Novel plant genotypes

Describe the methods by which all novel plant genotypes were produced. This includes those generated by transgenic approaches, gene editing, chemical/radiation-based mutagenesis and hybridization. For transgenic lines, describe the transformation method, the number of independent lines analyzed and the generation upon which experiments were performed. For gene-edited lines, describe the editor used, the endogenous sequence targeted for editing, the targeting guide RNA sequence (if applicable) and how the editor was applied.

Authentication

Describe any authentication procedures for each seed stock used or novel genotype generated. Describe any experiments used to assess the effect of a mutation and, where applicable, how potential secondary effects (e.g. second site T-DNA insertions, mosiacism, off-target gene editing) were examined.

Magnetic resonance imaging

Experimental design

Design type

resting-state fMRI

Design specifications

All participants were scanned on a 3-T Siemens connectome-Skyra scanner (customized to achieve 100 mT/m gradient strength) at Washington University in St. Louis.Each participant underwent two sessions with two 15-min resting-state scans per session, utilizing a 32-channel head coil. Scanning parameters were: TR = 720 ms, voxel size = 2 mm isotropic. This resulted in four 1,200 sampled time points for each participant. During the resting state, participants were instructed to fixate on a crosshair, remaining awake with eyes open. Simultaneous cardiac and respiratory signals were recorded using pulse oximetry on a finger digit and a belt sensor around the abdomen, respectively, time locked to fMRI scan onset at a sampling rate of 400 Hz.

Behavioral performance measures

We did a data exclusion and data reduction for the original set of 478 behavioral measures in the HCP young adult dataset. We excluded 359 and kept 119 variables as following criteria: 1) 105 bad variables that were quantitatively poor measures including having 100 standard deviations above the median, fewer than half valid values (i.e., 500) or same values exceeding 95% of the data. 2) 11 confounding variables including acquisition reconstruction software version, gender, age, weight, height, BMI, systolic blood pressure, diastolic blood pressure, Hemoglobin A1c measured in blood, the cube-root of total brain volume (including ventricles) and the cube-root of total intracranial volume. 3) uninterested variables in demographic measures. 4) redundant variables in cognition test and substance abuse retrospective. For more details, see supplementary material.

Acquisition

Imaging type(s)

resting state-functional MRI

Field strength

3 Tesla

Sequence & imaging parameters

2D multiband gradient echo-planar imaging sequence with TE=33.1ms, TR=720ms, flip angle=52°, 2.0mm isotropic voxels, 72 slices, multiband factor of 8.

Area of acquisition

whole brain

Diffusion MRI ☐ Used ☒ Not used

Preprocessing

Preprocessing software

Data pre-processing step was implemented using Workbench and custom codes in MATLAB 2020b.

Normalization

nonlinear registration of T1w images

Normalization template	MNI standard volumetric space
Noise and artifact removal	We primarily analyzed FIX-denoised data with supplementary analyses using the minimally-preprocessed data. The critical distinction between these approaches lies in ICA-FIX's capacity to remove spatially specific structured noise components, including: (1) spatial overlap with white matter, cerebrospinal fluid, or blood vessels; (2) signal localized at the edges of the brain (motion) or in areas of signal drop (susceptibility); (3) spatially ill-defined component clusters; (4) non-dominant low-frequency (<0.1 Hz) spectral power; and (5) transient signal spikes. Additional noise regression procedures were implemented through custom MATLAB code11. These included removal of linear trends for each run and regression of nuisance time series encompassing cerebrospinal fluid flow signals from ventricles, white matter signals, and head motion parameters.
Volume censoring	No volume censoring.

Statistical modeling & inference

Model type and settings	Canonical correlation analysis
Effect(s) tested	Canonical correlation coefficient and the corresponding weights of topography and behavior.

Specify type of analysis: ☒ Whole brain ☐ ROI-based ☐ Both

Statistic type for inference<br>(See Eklund et al. 2016)	5,000 permutation test
Correction	family-wise error rate

Models & analysis

n/a	Involved in the study
☒	☐ Functional and/or effective connectivity
☒	☐ Graph analysis
☐	☒ Multivariate modeling or predictive analysis

Multivariate modeling and predictive analysis	We performed principal component analysis to reduce dimensionality and canonical correlation analysis to investigate the relationship between brain and behavioral measures. Brain measures included global signal topography (Pearson's correlation between the global signal and fMRI time series), respiration topography (Pearson's correlation between respiration measures and fMRI time series), and cardiac topography (Pearson's correlation between cardiac measures and fMRI time series).
